# Percutaneous Thrombovegectomy as an Alternative to Surgery for Tricuspid Valve Endocarditis

**DOI:** 10.1016/j.atssr.2024.03.012

**Published:** 2024-04-26

**Authors:** V. Seenu Reddy, Brittany A. Zwischenberger, Adam R. Williams, Joseph F. Rowe, Sreekumar Subramanian, Adam Kingeter, Justin Wright, Mark Joseph

**Affiliations:** 1 Division of Cardiothoracic Surgery, TriStar Centennial Medical Center, HCA Healthcare, Nashville, Tennessee; 2 Department of Surgery, Duke University Medical Center, Durham, North Carolina; 3 Department of Surgery, Virginia Tech Carilion School of Medicine, Roanoke, Virginia; 4 Department of Anesthesiology, TriStar Centiennial Medical Center, HCA Healthcare, Nashville, Tennessee

## Abstract

**Background:**

Undergoing an urgent valve surgical procedure to treat patients with tricuspid valve endocarditis carries a high risk of operative morbidity and mortality. Use of a percutaneous vacuum-assisted system to treat tricuspid valve endocarditis is an alternative to surgical procedures.

**Methods:**

This study retrospectively analyzed data from 187 transcatheter vacuum-assisted aspiration procedures performed in 177 patients with tricuspid valve vegetations at 3 different centers between 2017 and April 2022. The device was deployed through the internal jugular or femoral vein into the right atrium by using transesophageal echocardiography and fluoroscopy guidance with the return cannula placed in the femoral vein. The following data were analyzed: intravascular material aspirated, collected in an external filter, and cultured; hospital length of stay; procedural complications; blood culture clearance; and tricuspid regurgitation.

**Results:**

The mean age of participants was 35.5 ± 10.8 years (range, 18-79 years). A total of 107 procedures (57.2%) were performed in female patients, and 163 (87.2%) procedures were performed in intravenous drug users. All patients survived the procedure, and there were no procedural complications. The average length of stay was 26.8 ± 18.5 days (range, 1-96 days). Most preoperative positive blood cultures showed *Staphylococcus aureus* (80.3%), with postoperative cultures converting to negative in 103 (70.1%) of 147 procedures. Tricuspid regurgitation remained unchanged after 95 (50.8%) procedures and worsened after 40 (21.4%) procedures.

**Conclusions:**

Percutaneous vacuum-assisted aspiration system provides a safe alternative to urgent tricuspid valve surgical procedures for removal of vegetations, especially in patients with endocarditis. Initial vegetation debulking can expedite clearance of blood cultures while avoiding major cardiac surgery operations and implantation of prosthetic valves in these high-risk patients.

**In Short**

▪ Percutaneous vacuum-assisted aspiration is a safe and effective option for removing tricuspid valve vegetations in patients with infective endocarditis, including intravenous drug users.
▪ The percutaneous vacuum assist system may be used as an alternative to surgical valve procedures for patients with right-sided infective endocarditis.

Right-sided infective endocarditis (RSIE) cases have increased in recent years in association with the sharp rise in opioid and heroin abuse.^1^ Intravenous drug users (IVDUs) who have infective endocarditis are more likely to be infected by *Staphylococcus aureus* with tricuspid valve involvement and are 3 times more likely to have surgery for RSIE compared with non-IVDUs.^1^^,^^2^

American Association for Thoracic Surgery consensus guidelines for the surgical treatment of infective endocarditis recommend surgical treatment for patients with heart failure, severe valve dysfunction, prosthetic valve endocarditis, recurrent systemic embolization, large mobile vegetations, and persistent sepsis despite adequate antibiotic therapy for more than 5 to 7 days.^3^ Although many patients with RSIE with small lesions can be managed with medical therapy, 15% to 30% of patients with RSIE require surgical intervention.^4^ Surgical treatment of tricuspid valve infective endocarditis is associated with a 6% to 7% mortality rate and a high likelihood of postoperative morbidity with prolonged intensive care unit and hospital stays.^5^ Pain management is also complex and may lead to ongoing opioid dependence.

IVDUs undergoing surgical valve procedures for infective endocarditis have a 29% to 59% increase in length of stay and 26% to 41% higher hospital charges compared with non-IVDUs.^1^^,^^6^ Schranz and colleagues^1^ reported that total charges for patients with infective endocarditis hospitalizations requiring surgical treatment were significantly higher for IVDUs, with a median charge of $250,994 compared with $198,764 for non-IVDUs. IVDUs are also 3 times more likely to be underinsured or uninsured compared with non-IVDUs.^1^

The use of a percutaneous vacuum-assisted aspiration system (AngioVac System, AngioDynamics) to remove tricuspid valve vegetations has recently emerged as a potential alternative to surgical valve procedures in patients with RSIE.^7^ This system enables minimally invasive percutaneous, right-sided valve vegetation removal through suction filtration and venovenous bypass, thereby eliminating the risk and prolonged recovery associated with surgical treatment. Recently published European Society of Cardiology guidelines for managing endocarditis added debulking of right intraatrial septic masses by aspiration for patients at high surgical risk.^8^ The objective of the present study was to provide real-world insights on the use of the percutaneous vacuum-assisted procedure for debulking tricuspid valve vegetations in patients with tricuspid valve endocarditis.

## Patients and Methods

This study is a multicenter, retrospective analysis of consecutive procedures using a percutaneous vacuum-assisted aspiration system in patients with tricuspid valve endocarditis between 2017 and April 2022. A waiver for obtaining patient consent was approved by the Institutional Review Boards at TriStar Centennial Medical Center (Nashville, TN), Carilion Clinic (Roanoke, VA), and Duke University (Durham, NC). Patients included in the study had indications for surgical treatment of tricuspid valve endocarditis extending into the right atrium or ventricle, active bacteremia unresponsive to antibiotics, or evidence of septic pulmonary emboli. Perioperative data were extracted from medical records. Quantitative variables are presented as mean + SD, and categoric variables are summarized as percentages.

### Interventional Technique

Use of the percutaneous vacuum-assisted aspiration system has been previously described.^9^ The procedures were performed most commonly in a hybrid operating room suite, a traditional operating room, or a cardiac catheterization laboratory with the patient general anesthesia and with perfusion team support and continuous transesophageal echocardiography (TEE). The system’s 22-F inflow cannula with a balloon actuated (Gen2) or an expandable funnel shaped, angled distal tip (Gen3), and its dilator, were inserted in the right internal jugular vein or femoral vein over an extrastiff guidewire and advanced into the right atrium under TEE and fluoroscopic guidance. A return cannula was placed in the common femoral vein under ultrasound guidance on the basis of patients’ anatomy and institutional preference. The cannulas were “wet” connected to the extracorporeal circuit with an inline 170-μm bubble trap filter (Terumo). Venovenous extracorporeal bypass was started with a flow of 2500 to 3000 cc/min. The inflow funnel tip cannula was then passed in proximity to and across the tricuspid valve and its annulus several times to remove the vegetation under TEE and fluoroscopic guidance. Once debulking of the intracardiac lesions was complete, the circuit was discontinued, and filtered blood was returned to the patient, the filter was flushed, and the cannulas were removed. The aspirated vegetation collected in the filter was sent for culture and pathologic evaluation. Procedural success was defined as removal of most (>90%) debulking of vegetation determined by TEE. Patients were followed up postoperatively on an outpatient basis at various intervals with repeat echocardiography to assess recurrent valve disease, valve regurgitation, and signs and symptoms of right-sided heart dysfunction.

## Results

### Preoperative Patient Characteristics

A total of 187 procedures were performed in 177 patients at 3 centers during the study period. Eight patients underwent 2 separate procedures, and 1 patient had 3 procedures with the device during separate admissions. Baseline patient and clinical characteristics are summarized in Table 1. The mean patient age was 35.5 ± 10.8 years. A total of 163 (87.2%) of the procedures were performed in patients who were IVDUs (Supplemental Table 1). For the 147 procedures where insurance coverage data were available, most (76.0%) either were covered by Medicaid (45.9%) or the patient was uninsured (30.1%).

**Table 1:** Patient Demographics and Procedural Outcomes Data

Characteristic	Number of procedures
Sex	
Male	80 (42.8)
Female	107 (57.2)
Mean age ± SD, y	35.5 ± 10.8 (18-79)
Insurance coverage	
Commercial	28 (19.2)
Medicare	7 (4.8)
Medicaid	67 (45.9)
Uninsured	44 (30.1)
Missing	41 (28.1)
Valve vegetation size ± SD, cm	2.15 ± 0.91
Required postprocedure valve surgery	3 (1.6)
Mean length of hospital stay ± SD, d	26.0 ± 18.1 (3-96)
Postprocedure sterilization of blood cultures^a^	103 (70.1)
Postprocedure valve vegetation size ± SD, cm	0.59 ± 0.85

Values are n (%) or mean ± SD (range).

a For patients with positive preprocedural blood culture results.

**Figure 1:**
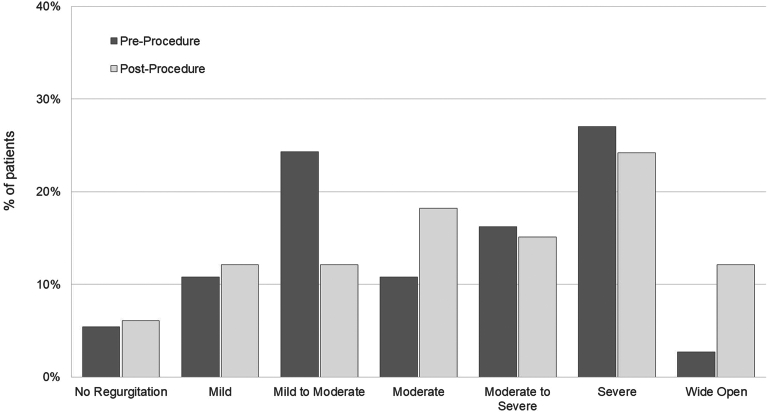
Comparison of preprocedural and postprocedural tricuspid regurgitation.

Positive preoperative blood culture results were recorded for 147 (78.6%) patients (Supplemental Table 2). The most common organism was *Staphylococcus* species alone or in combination with another microorganism in 123 of 147 (83.7%) of the cultures. Methicillin-resistant *Staphylococcus aureus* was presented in 56 (38.1%) of the positive cultures. Mean preoperative valve vegetation size was 2.15 ± 0.91 cm. The degree of preoperative tricuspid valve regurgitation across the patient population is shown in Figure 1.

### Outcomes Data

The average length of stay was 26.8 ± 18.5 days. One patient experienced a postoperative pericardial effusion requiring drainage through a subxiphoid window, and the effusion resolved before discharge. Another patient had an embolism to the pulmonary artery and required inotropic support. Although the patient was extubated and removed from inotropic therapy, this patient ultimately died of multiorgan system failure. A second postoperative death occurred in a patient who experienced respiratory failure.

Three patients underwent valve surgery. In 1 of these patients, the procedure was performed during the initial hospital admission as a result of involvement of a left-sided heart valve (mitral) requiring valve replacement. The 2 other patients underwent subsequent tricuspid valve replacement after demonstrating symptoms of right-sided heart failure.

Postprocedural sterilization of blood cultures occurred after 103 (70.1%) of the 147 procedures where preoperative positive blood cultures tested positive. Postprocedural vegetation size was documented for 109 (58.3%) of the procedures, with a mean postoperative vegetation size of 0.59 ± 0.85 cm, equating to a 72.5% reduction vs the mean preoperative vegetation size. Postprocedural echocardiography demonstrated that tricuspid regurgitation improved after 28 (15.0%) procedures, remained unchanged after 95 (50.8%) procedures, and worsened after 40 (21.4%) procedures. No echocardiographic data were available for the remaining 24 (12.8%) procedures.

## Comment

Several recent studies and case reports have reported on the use of the percutaneous vacuum-assisted system to remove tricuspid valve vegetations in patients with RSIE. Although these reports focused on the feasibility of this approach for removal of tricuspid valve vegetations, they were limited to smaller patient populations from single centers.^7^ The present study reports on a much larger group of patients treated at 3 different centers and represents a large published clinical experience on the use of this device for patients with valvular endocarditis. We were able to demonstrate that the use of this device to treat RSIE was both safe and effective, with only 3 patients requiring subsequent surgical tricuspid valve replacement.

We hypothesize that the reduction in vegetation size we observed led to a reduction in bacterial load that improved the effectiveness of subsequent antibiotic therapy. This concept is supported by our findings that 70% of patients who underwent the procedure and had positive blood culture results preoperatively subsequently had negative blood culture results shortly after the procedure during their inpatient stay. As discussed by Abubakar and colleagues,^9^ removal of valvular vegetation by using the percutaneous vacuum-assist device may reduce antibiotic resistance, as has been observed with high bacterial inocula leading to an enhancement of antibiotic activity.

Although 21.4% of the procedures in our series were associated with postprocedural worsening of tricuspid valve regurgitation before discharge, this tricuspid valve dysfunction was well tolerated, with no additional intervention required*.* Importantly, the implantation of a bioprosthetic valve in a patient with positive blood culture results was avoided, thus potentially reducing the rates of prosthetic valve endocarditis.

Of note is that most of the patients in our series (87.2%) were IVDUs. This patient population experiences longer hospital stays and increased costs compared with non-IDVUs when undergoing surgical valvular procedures.^1^ We demonstrated in the present study that the percutaneous vacuum-assisted system offers a nonsurgical alternative that can assist in avoiding the need for urgent or acute valve surgery in nearly all patients treated with this procedure. In addition, because residual tricuspid regurgitation was well tolerated, this procedure provided time for these patients to be treated more completely for their underlying substance abuse and other comorbidities.

**Figure 2:**
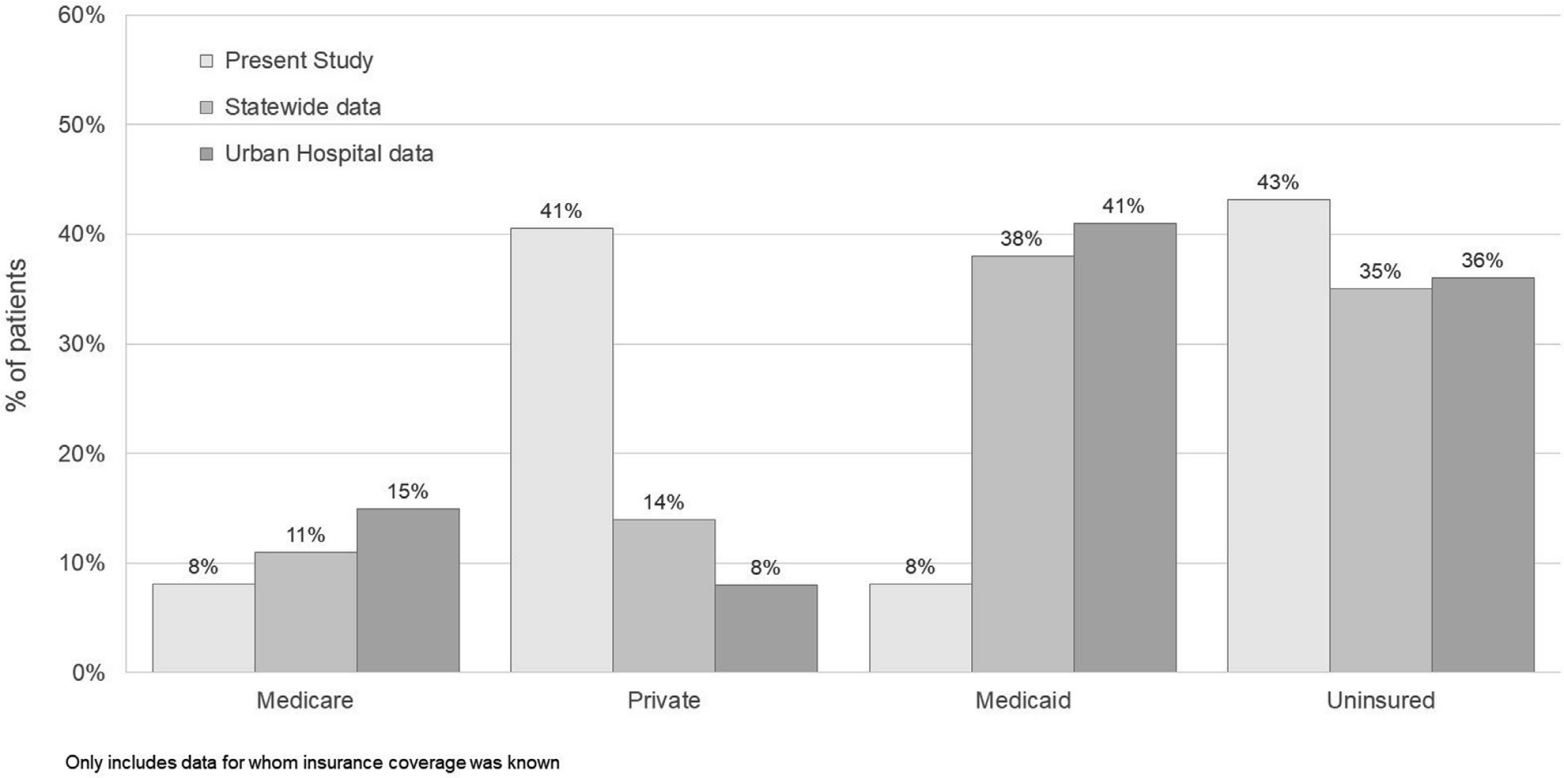
Comparison of insurance coverage for intravenous drug users with infective endocarditis in the current study vs other reports. Data from^1^^,^^7^.

A large percentage of the IVDUs in our study had no insurance coverage (Figure 2). The ability to avoid an open surgical procedure in patients with infective endocarditis has a large potential impact on hospital economics, especially for patients with limited or absent insurance coverage. Table 2 provides estimated differences in hospital costs for patients undergoing valve surgery compared with the use of the percutaneous vacuum-assisted device on the basis of published hospital charge data for patients with infective endocarditis.^1^ This analysis assumes an incremental per procedure cost beyond the cost of managing the patient medically of $30,392. This includes the following: the percutaneous vacuum-assisted system cost; procedural supply expenses; intensive care, room, and staff costs; and costs associated with conversion to a surgical procedure, when required. Hospital charges were converted to costs by using a 32% cost-to-charge ratio, the median cost-to-charge ratio for US hospitals as reported by Medicare.^6^ On the basis of our model’s assumptions, there is an estimated cost savings of $38,932 associated with the use of the percutaneous vacuum-assist system vs valve surgery for IVDUs with infective endocarditis and a savings of $33,213 for non-IVDUs.

**Table 2:** Estimated Differences in Hospital Costs: Valve Surgery vs Percutaneous Vacuum-assisted Procedure

Patient Population	Median Hospital Charges, $	Estimated Median Hospital Costs,^a^ $
Intravenous drug users		
All patients (medical and surgical treatment)^b^	60,333	19,307
Surgical treatment^b^	250,994	80,318
Medical treatment^c^	36,647	10,994
Estimated cost of percutaneous vacuum-assist procedure^d^	…	30,392
Estimated total cost of hospital stay for patients undergoing percutaneous vacuum-assist procedure	…	41,386
Cost differential: surgical valve vs percutaneous vacuum-assist procedure	…	38,932
Non–intravenous drug users		
All patients (medical and surgical treatment)^b^	34,968	11,190
Surgical treatment^b^	198,764	63,605
Medical treatment^c^	11,282	3610
Estimated cost of percutaneous vacuum assist procedure^d^	…	30,392
Estimated total cost of hospital stay for patients undergoing percutaneous vacuum-assist procedure	…	41,386
Cost differential: surgical valve vs percutaneous vacuum-assist procedure	…	33,213

a Estimated using 32% cost-to-charge ratio^6^

b Data from^1^

c Assumes that 8.6% of patients with infective endocarditis undergo surgical treatment^1^

d Includes estimated percutaneous vacuum-assisted system cost, procedural supplies, room and staff costs, and costs associated with conversion to a surgical procedure, when required.

Important limitations of our study include the lack of a control group and the use of a retrospective study design that introduces the potential for bias relative to the patients selected to undergo this procedure and limits the ability to identify clearly those patients who may potentially benefit from this approach. A well-designed, prospective study (in comparison with surgical or medical therapy) would be required to determine answers to the foregoing conclusively. Although the estimates of a potential cost difference between the percutaneous vacuum-assisted procedure and valve surgery we report are not based on actual cost data for the patients in our series, our modeling can serve as a basis for conducting a similar analysis in differing geographic or institutional settings.

In conclusion, the present study describes an available percutaneous approach for the treatment of patients with RSIE that may provide a safer, more effective alternative to urgent surgical removal of tricuspid valve vegetations, with fewer complications and faster recovery. Further research and clinical studies are needed to validate the benefits associated with this approach.
