# Supplementary material for: Percutaneous Thrombovegectomy as an Alternative to Surgery for Tricuspid Valve Endocarditis
Source: Ann Thorac Surg Short Rep. 2024 Apr 26;2(4):748–53. doi: 10.1016/j.atssr.2024.03.012 (PMC11708743; doi:10.1016/j.atssr.2024.03.012)
Supplement: Supplemental Tables [file mmc1.pdf]

Supplemental Materials

Percutaneous Thrombo-Vegectomy as an Alternative to Surgery for Tricuspid Valve Endocarditis

Supplemental Table 1. Etiology of endocarditis

Etiology	# of procedures (%)
Intravenous drug use	163 (87.2%)
Venous catheter	9 (4.8%)
Cardiac lead	4 (2.1%)
Pneumonia	2 (1.1%)
Hip infection	1 (0.5%)
Prosthetic knee infection	1 (0.5%)
Osteomyelitis of the foot	1 (0.5%)
Tooth infection	1 (0.5%)
Myxoma	1 (0.5%)
Right atrial mass	1 (0.5%)
End-stage renal disease/septic arthritis	1 (0.5%)
Missing	2 (1.1%)

Supplemental Table 2. Blood culture results

Characteristic	# of procedures (%)
Positive blood cultures	147 (78.6%)
Organisms cultured	
MSSA	57 (30.5%)
MRSA	56 (29.9%)
Enterococcus	5 (2.7%)
Staphylococcus aureus	5 (2.7%)
MSSA and Candida	2 (1.1%)
Candida	2 (1.1%)
Klebsiella	2 (1.1%)
Staphylococcus hominis	2 (1.1%)
Gram (+) cocci, undefined	2 (1.1%)
Serratia marcescens	2 (1.1%)
Alpha Hemolytic Streptococcus	1 (0.5%)
Coagulase Negative Staphylococcus	1 (0.5%)
Enterobacter Cloacae	1 (0.5%)
Gram (-) rod, undefined	1 (0.5%)
Haemophilus influenzae	1 (0.5%)
Streptococcus mitis / Neisseria sicca	1 (0.5%)
Staphylococcus aureus / Streptococcus oralis / Serratia marcescens	1 (0.5%)
Staphylococcus aureus / Enterobacter	1 (0.5%)
Streptococcus	1 (0.5%)
Streptococcus mitis / Streptococcus anginosus / Staphylococcus hominis	1 (0.5%)
Streptococcus viridans	1 (0.5%)
Missing	1 (0.5%)

MRSA = Methicillin-resistant *Staphylococcus aureus*

MSSA = Methicillin-sensitive *Staphylococcus aureus*
